# The Role of Probiotics in Alleviating Postweaning Diarrhea in Piglets From the Perspective of Intestinal Barriers

**DOI:** 10.3389/fcimb.2022.883107

**Published:** 2022-05-30

**Authors:** Weifa Su, Tao Gong, Zipeng Jiang, Zeqing Lu, Yizhen Wang

**Affiliations:** ^1^ National Engineering Laboratory of Biological Feed Safety and Pollution Prevention and Control, Zhejiang University, Hangzhou, China; ^2^ Key Laboratory of Animal Nutrition and Feed, Ministry of Agriculture, Zhejiang University, Hangzhou, China; ^3^ Key Laboratory of Molecular Animal Nutrition, Ministry of Education, Zhejiang University, Hangzhou, China; ^4^ Key Laboratory of Animal Nutrition and Feed Science of Zhejiang Province, Institute of Feed Science, Zhejiang University, Hangzhou, China

**Keywords:** piglets, postweaning diarrhea, antibiotics, probiotics, intestinal barriers

## Abstract

Early weaning of piglets is an important strategy for improving the production efficiency of sows in modern intensive farming systems. However, due to multiple stressors such as physiological, environmental and social challenges, postweaning syndrome in piglets often occurs during early weaning period, and postweaning diarrhea (PWD) is a serious threat to piglet health, resulting in high mortality. Early weaning disrupts the intestinal barrier function of piglets, disturbs the homeostasis of gut microbiota, and destroys the intestinal chemical, mechanical and immunological barriers, which is one of the main causes of PWD in piglets. The traditional method of preventing PWD is to supplement piglet diet with antibiotics. However, the long-term overuse of antibiotics led to bacterial resistance, and antibiotics residues in animal products, threatening human health while causing dysbiosis of gut microbiota and superinfection of piglets. Antibiotic supplementation in livestock diets is prohibited in many countries and regions. Regarding this context, finding antibiotic alternatives to maintain piglet health at the critical weaning period becomes a real emergency. More and more studies showed that probiotics can prevent and treat PWD by regulating the intestinal barriers in recent years. Here, we review the research status of PWD-preventing and treating probiotics and discuss its potential mechanisms from the perspective of intestinal barriers (the intestinal microbial barrier, the intestinal chemical barrier, the intestinal mechanical barrier and the intestinal immunological barrier) in piglets.

## Introduction

As a critical period, the health of piglets during the weaning period determines later growth performance (Gresse et al., 2017). In the modern porcine industry, early weaning generally occurs at 3-4 weeks of age to improve economic efficiency (Sutherland et al., 2014). Nevertheless, the digestive and immune systems of piglets are immature at this stage. Study has shown that the activities of piglets’ digestive enzymes, such as pepsin, trypsin, chymotrypsin and amylase, significantly decreased within 1 week of early weaning, making feed difficult to digest (Jensen et al., 1997). Simultaneously, the change of feed from liquid milk to solid feed results in the destruction of intestinal physical barrier, including the destruction of tight junctions (TJs), reducing mucin production, and increasing of gut permeability, etc. (Le Dividich and Seve, 2000; Hu et al., 2013; Wang et al., 2016a). Additionally, early weaning causes the loss of microbial diversity and dysbiosis of gut microbiota, and further increases the risk of gastrointestinal diseases of piglets (Guevarra et al., 2018). Studies have reported that weaning transition reduces the relative abundance of *Lactobacillus* (the primary gut microbiota in piglet shaped by the sows’ milk), increases the relative abundance of *Clostridium* spp., *Prevotella* spp., *Proteobacteriaceae*, and *E. coli* (Konstantinov et al., 2006). Early weaned piglets are susceptible to enterotoxigenic *E. coli* (ETEC) infection and causing PWD, which kills up to 50% of piglets worldwide each year (Gresse et al., 2017).

In modern farming, antibiotics are heavily used to prevent and treat pig diseases in order to reduce economic losses. (Li, 2017). The long-term overuse of antibiotics is a screening process of bacteria and accelerates the spread of drug-resistant bacteria in animal gastrointestinal tract (Pamer, 2016). Such as, ETEC shows significant high resistance in porcine intestinal tract (Laird et al., 2021). While the gut microbial ecosystem is normally resilient, the composition of gut microbiota is relatively simple in the newborn mammals, resulting in a low resilience of the gut microbiota. The use of antibiotics permanently changes the structure of the microbial community and interferes with the intestinal homeostasis of newborn mammals (Sommer et al., 2017; Zong et al., 2020). Furthermore, studies have shown that antibiotics promote intestinal inflammation (Zeng et al., 2017), and antibiotics are associated with the decrease of microbiota diversity, exacerbating the vicious circle of PWD (Perez-Cobas et al., 2013). Antibiotics can also remain in the bodies of livestock, ultimately affecting human health. Therefore, antibiotics have been forbidden to be fed on livestock in many countries and regions. As the world’s largest pig farming country, since 1 July 2020, China have started to ban the feed production enterprises to product commercial feed containing growth-promoting drugs feed additives. Hence, there is an urgent need for developing nonantibiotic alternative to restore microbial balance and control PWD of piglets. The effects of probiotics, an alternative to antibiotics, on treating PWD are widely documented in recent years (**Table 1**). The most frequently used microorganisms are *Lactobacillus*, *Bifidobacterium*, *Enterococcus*, *Bacillus* and yeasts from the genus *Saccharomyces* (Liao and Nyachoti, 2017). A comprehensive understanding of the interactions between probiotics and intestinal barrier of piglets during PWD will help develop new probiotics interventions strategies that can enhance piglets’ growth performance and protect piglets from PWD.

**Table 1:** Effects of probiotics on treating PWD of piglet.

Microorganism Category	Microorganism Name	Treatment	Host Health Influence	Reference
Lactic acid bacteria	*Lactobacillus acidophilus, Lactobacillus casei, Bifidobacterium thermophilum and Enterococcus faecium*	Piglets weaned at 28 d of age were fed the basal diet mixed the probiotics (0.25 × 10^8^ CFU/g for each strain) for 25 days, and orally administered with ETEC F18+ (2 × 10^9^ CFU/g) on day 13 postweaning	Decreasing serum TNF-α; increasing jejunal villus height, and especially villus height-to-crypt depth ratio in piglets	(Sun et al., 2021b)
	*Lactobacillus delbrueckii*	The piglets were orally administrated with *Lactobacillus delbrueckii* (50 × 10^8^ CFU/mL) at amounts of 1, 2, 3, and 4 mL per animal at 1, 3, 7, and 14 d of age	Increasing the height of intestinal villi of piglets; promoting the expression of intestinal TJs proteins, and reducing the incidence of diarrhea by more than 50%	(Li et al., 2019b)
	*Enterococcus faecalis*	Piglets weaned at 26 d of age were fed basal diet supplemented with *Enterococcus faecalis* (2.5 ×10^9^ CFU/kg) for 28 days	*Enterococcus faecalis* and neomycin sulfate decreased diarrhea index and improve growth performance, *Enterococcus faecalis* increased *Lactobacillus* in feces	(Hu et al., 2015)
	*Lactobacillus plantarum*	Piglets (4 d of age) were orally administrated with* Lactobacillus plantarum* (5 × 10^10^ CFU/kg) for 15 days and then orally administrated with ETEC F4 (1 × 10^8^ CFU per pig)	Improving performance and effectively preventing the diarrhea; improving function of the intestinal barrier by protecting intestinal morphology and intestinal permeability and the expression of genes for TJs proteins	(Yang et al., 2014)
	*Lactobacillus zeae* and *Lactobacillus casei*	Piglets weaned at 28 d of age were fed corn-soybean meal mixed feed fermented by *Lactobacillus zeae* and *Lactobacillus casei* for 3 days, and then orally challenged with 1 mL *Salmonella* (1 × 10^6^ CFU/mL)	Decreasing pro-inflammatory cytokine expression and alleviating *Salmonella* infection	(Yin et al., 2014)
Yeast	*Saccharomyces cerevisiae*	Piglets weaned at 14 d b of age were fed basal diet supplemented with 3.0 g kg^–1^ live yeast *Saccharomyces cerevisia*e (4.3 × 10^9^ CFU/g) for 21days	Decreasing numbers of *Escherichia coli* in the ileum and cecum contents; increasing serum SOD activity and jejunum mucosal SIgA secretions	(Zhu et al., 2017a)
	*Saccharomyces cerevisiae*	Piglets weaned at 28 d of age were fed basal diet supplemented with 5 g/kg live yeast *Saccharomyces cerevisia*e for 14 days, orally challenged with ETEC F4 (1.5 × 10^11^ CFU/piglet) after weaning (d 29)	Significantly lower daily diarrhea scores, duration of diarrhea, and shedding of pathogenic ETEC bacteria in feces and increasing IgA levels in the serum of piglets	(Trckova et al., 2014)
	*Saccharomyces cerevisiae*	Piglets weaned at 21 d of age were fed basal diet supplemented with *Saccharomyces cerevisiae* fermentation products for 8 days and then orally challenged with ETEC F4	*Saccharomyces cerevisiae* fermentation products and carbadox increased average daily feed intake, *Saccharomyces cerevisiae* fermentation products decreased the ileal mucosa adherent *Escherichia coli* ETEC F4	(Kiarie et al., 2011)
	*Saccharomyces cerevisiae* var. *boulardii*	Piglets weaned at 26 d of age were fed basal diet supplemented with 200 g/t live *Saccharomyces cerevisiae* var. *boulardii* for 16 days, and then dosed *via* indwelling jugular catheters with *Escherichia coli* lipopolysaccharide (LPS) (25 μg/kg of BW)	ADG increased by 39.9% and LPS-induced piglet mortality was reduced 20%	(Collier et al., 2011)
	*Saccharomyces cerevisiae*	Piglets weaned at 21 d of age were fed *Saccharomyces cerevisiae* fermentation products for 14 days, and then orally administrated with *Salmonella* (1 × 10^9^ CFU)	Increasing compensatory body weight gains after *Salmonella* infection and increasing *Salmonella* shedding in feces	(Price et al., 2010)
*Bacillus*	*Bacillus subtilis* KN-42	Piglets weaned at 28 d of age were fed basal diet supplemented with 20 × 10^9^ CFU/kg feed of *B. subtilis* KN-42 for 28 days	*Bacillus subtilis* KN-42 increased average daily gain (ADG) and feed efficiency of piglets, *Bacillus subtilis* KN-42 and neomycin sulfate decreased diarrhea index and the relative number of *Escherichia coli*	(Hu et al., 2014)
	*Clostridium butyricum*	Piglets (7.09 ± 0.2 kg) were fed basal diet supplemented with *Clostridium butyricum* (5 × 10^5^ CFU/g) for 15 days and then orally administered with ETEC F4 (1 × 10^9^ CFU/g)	Alleviating intestinal villi injury caused by ETEC F4 challenge	(Li et al., 2021)
Three types of mixed bacteria	*Enterococcus faecium*, *Bacillus subtilis*, *Saccharomyces cerevisiae and Lactobacillus paracasei*	Piglet weaned at 28 d of age were fed the basal diet mixed the probiotics (>1 × 10^8^ CFU/g for each strain) for 21days	Increasing fecal acetic acid and propionic acid; increasing growth performance and significantly reducing PWD	(Lu et al., 2018)

## Intestinal Barriers of Piglets

The intestinal barriers of piglets are consisted of microbial barrier, mucosal barrier and immunological barrier. The intestinal barriers play an important role in maintaining the homeostasis of the gut internal environment (Maynard et al., 2012). As a critical line of defense, intestinal barrier prevents the pathogenic antigens, toxins and pathogenic microorganisms from invading the internal environment of the body (Baumgart and Dignass, 2002).

Newborn piglets develop a diverse and complex microbial community in the gastrointestinal tract by milk intake and exposure to the external environment (Blaut and Clavel, 2007). The dynamic balance, formed by interdependence and mutual restraint among different gut microbiota, provides the first barrier for gut. In the face of the external threats, the gut microbiota works together to counteract its own disadvantages (Hooper and Macpherson, 2010; Shanahan, 2010). There are three widely accepted mechanisms of gut microbial barrier function: 1) occupying the binding site and settlement space; 2) nutrition competition; 3) promoting the improvement of intestinal function (regulating the secretion of mucus and the development of intestinal immune system) (Buffie and Pamer, 2013; Kamada et al., 2013).

The mucosal barrier, the second intestinal barrier in piglets, consists of chemical and mechanical barriers (Sperandio et al., 2015). Chemical barrier is composed of the mucus secreted by the intestinal mucosa epithelium, digestive liquid, and bacteriostatic substances produced by normal parasitic bacteria in the intestinal lumen. Paneth cells and goblet cells contribute to the natural immune defense that supports epithelial barrier function (McCracken and Lorenz, 2001). Paneth cells produce antimicrobial agents such as defensins and lysozyme, and they can damage bacterial cell walls or membranes to inhibit or kill pathogenic bacteria and maintain gut mucosal homeostasis (Salzman et al., 2007; Bevins and Salzman, 2011; Salzman, 2011; Sun et al., 2021a). Additionally, the mucin, produced by goblet cells, forms a protective layer to prevent pathogenic microbes from binding to intestinal epithelial cells (Desai et al., 2016). The gut microbiota and the host immune cells can ingeniously modulate these barriers to avoid unnecessary immune responses to gut commensal microbes by spatially segregating the gut microbiota and the host immunity (Okumura and Takeda, 2018). The structure of mechanical barrier is based on intact intestinal epithelial cells (ICEs) and TJs between epithelial cells. ICEs and TJs can effectively prevent bacteria and endotoxins from entering the blood from intestine (Wang et al., 2014; Balda and Matter, 2016; Zong et al., 2021).

Early weaning is a challenge to the immature gut immune system as it must adapt to gut microbial colonization and feed antigens. In the early weaning period, the innate immune system defenses responsible for barrier function are more mature compared to the adaptive immune system. Therefore, the early weaning piglet is more reliant on innate immunity (Humphrey et al., 2019). The intestinal immune system is stimulated to maintain homeostasis in the intestinal epithelium by secreting immunoglobulins, interleukins and interferons. At approximately 6 weeks of age, piglets have stable numbers of lymphocytes and mature secondary lymphoid organs, such as Peyer’s patches (PPs) in the gut (Moeser et al., 2017). PPs are covered by a specialized follicle associated epithelium containing M cells, which is a pathway for antigens to enter the lamina propria. The lamina propria contains a variety of immune cells, mainly including B cells, macrophages, dendritic cells (DCs) and T cells (Allaire et al., 2019). The perception of microbes by epithelial cells, DCs and macrophages is mediated by pattern recognition receptors (PRRs) such as toll-like receptors (TLRs) (Xiao et al., 2017). The T cells closely related to probiotics in the lamina propria are T helper (Th) and regulatory T (T_Reg_) cells. The activation of PRRs often induces microbial killing pathways and activates T helper 1 (Th1) and T helper 17 (Th17) cells and adaptive immune cells (Liew, 2002).

## Probiotics Relieve PWD by Regulating the Intestinal Microbial Barrier

Probiotics can improve the richness of gut microbiota and shape the gut microbiota oriented by beneficial bacteria to resist infection by pathogenic microorganisms (Tang et al., 2020). Study has shown that supplementation with S. cerevisiae and Bacillus licheniformis reduced diarrhea incidence and the relative abundance of intestinal E. coli, and increased the relative abundance of Lactobacillus in ETEC-challenged piglets (Pan et al., 2017). Several recent studies suggested that dietary supplementation of lactic acid bacteria (Lactobacillus johnsonii, Lactobacillus plantarum, Lactobacillus delbrueckii and Enterococcus faecalis) increased the relative abundance of Lactobacillus or Bifidobacterium spp., decreased E. coli and enhanced production of short-chain fatty acids (SCFAs) in the gut of weaning piglets (Wang et al., 2019a; Wang et al., 2019b; Xin et al., 2020; Wang et al., 2021). This probiotic-mediated increase of SCFAs in the gut contributes to defend against pathogenic microbial invasion by downregulating the pH of the gastrointestinal tract, and enhances gut barrier function by providing energy to intestinal epithelial cells (Guilloteau et al., 2010; D'Souza et al., 2017). In addition, intestinal inflammation caused by PWD often leads to increased oxygen in the piglet intestine, which provides proliferation conditions for the facultative anaerobe, such as *Escherichia coli* (E. coli) (Wei et al., 2017). The increase of E. coli usually exacerbates PWD and creates a vicious cycle. Some aerobic probiotics (such as *Bacillus subtilis*) or facultative anaerobic probiotics (such as *Saccharomyces cerevisiae*) rapidly consume oxygen upon entering into the intestine, creating an anaerobic environment that inhibits the growth of aerobic pathogens in the gut (Hillman et al., 1994; Han et al., 2012).

Piglets are sensitive to pathogen colonization of the intestinal tract during weaning (Dubreuil, 2017). ETEC causes PWD in piglets mainly through intestinal mucosa adhesion, colonization and toxin production. Probiotics can exclude pathogens from attaching to mucosal surfaces by competition for shared binding sites and steric hindrance of protein adhesins of pathogenic bacteria (Nair et al., 2017; Yang et al., 2018). Wang et al. (2018) suggested that *Lactobacillus plantarum* inhibited the adhesion of ETEC to IPEC-J2 cells in a dose-dependent manner. Collado et al. (2007) found that *Bifidobacterium lactis* and *Lactobacillus rhamnosus* inhibited the adhesion of *Salmonella*, *Clostridium* and *E. coli* to pig intestinal mucus. *Saccharomyces cerevisiae* var. *boulardii* and β-galactomannan also inhibited *in vitro* adhesion of ETEC on cell surface of porcine intestinal IPI-2I cells (Badia et al., 2012a). In addition to inhibiting the adhesion of pathogens to the intestinal mucosa through competitive exclusion, probiotics also can secrete antimicrobial substances, such as bacteriocins, organic acids and hydrogen peroxide (Dicks and Botes, 2010; O'Shea et al., 2012; Knaus et al., 2017). The antimicrobial compounds exert direct antimicrobial effect against competing entero-pathogens and prevent the pathogenic colonization in the gastrointestinal tract of piglets (van Zyl et al., 2020). Moreover, *Bifidobacterium* was reported to bind and neutralize lipopolysaccharides (LPS) or Vero cytotoxin from *E. coli* (Kim et al., 2001; Park et al., 2007).

Therefore, the regulatory effects of probiotics to alleviate PWD of piglet through the intestinal microbial barrier mainly include the following three aspects: 1) shaping the gut microbiota oriented by beneficial bacteria; 2) competitive exclusion of pathogen; 3) producing antimicrobial substances. From the research status on the regulatory effect of probiotics on the intestinal microbial barrier of postweaning piglets, probiotics appear to be more effective in preventing PWD than in treating PWD. Screening for probiotics (such as *Lactobacillus*) that can stably colonize the piglet’s gut, efficiently produce antibacterial substances and competitively exclude of pathogen to prevent PWD may be an effective strategy. In addition, supplementing probiotics to accelerate the maturation of gut microbiota or to shape a PWD-preventing gut microbiota in weaned piglets are worthy of further study.

## Probiotics Relieve PWD by Regulating the Intestinal Chemical and Mechanical Barrier

The mucus layer in the intestine acts as the gatekeeper, separating the luminal microbiota from the epithelial cells (Birchenough et al., 2015; Zong et al., 2019a). Carvalho et al. (2012) reported that ETEC degraded MUC2 by secreting a serine protease (Eat A), which enabled bacteria to penetrate the mucus layer to reach the epithelium and triggered an inflammatory response (Kumar et al., 2014). Zhang et al., (Zhang et al., 2017) suggested that *Bacillus licheniformis-b* and *Bacillus subtilis* up-regulated the expression of *Atoh1* in the ileum of weaned piglets, which increased goblet cells number and MUC2 to protect the mucus barrier from the degradation of ETEC. *Lactobacillus reuteri* also enhanced intestinal mucosal barrier with the increase of goblet cells and antimicrobial peptides (AMPs) expressions of *MUC2*, *Lyz1*, and *pBD1* of piglets. Liu et al. (2017) reported that *Lactobacillus reuteri* increased the expression of porcine β-Defensin2 (*PBD2*), *pBD3*, *pBD114*, *pBD129* in the IPEC-J2 cells and colon of piglets. Similarly, Fu et al. (2021) reported that piglets treated by *Clostridium butyricum* or *Bacillus licheniformis* up-regulated the gene expression of *pBDs* and *PR-39* in jejunum. In another study, the bacterial secretory circular peptide and gassericin A of *Lactobacillus gasseri* LA39 and *Lactobacillus frumenti* can combine with Keratin 19 on the plasma membrane of intestinal epithelial cells to promote the fluid absorption and secretion reduction, thereby reducing the diarrhea of piglets (Hu et al., 2018b).

Many studies reported that probiotics relieved PWD by modulating the gut mechanical barrier. Yang et al. (2014) suggested that *Lactobacillus plantarum* alleviated the increase of urine lactic acid and plasma concentration and the decrease of *ZO-1* and *Occludin* mRNA and protein in the jejunum of piglets caused by ETEC. Hu et al. (2018a) demonstrated that oral administration of *Lactobacillus frumenti* can significantly improve the intestinal integrity and up-regulate the intestinal TJs proteins (ZO-1, Occludin, and Claudin-1) of piglets. Similarly, *Clostridium butyricum*, *Bacillus licheniformis* and *Lactobacillus reuteri* compete with potential pathogens for intestinal epithelial binding sites to promote TJs proteins expression (Li et al., 2019b; Zhao et al., 2019; Zong et al., 2019b). Study showed that *Lactobacillus plantarum* reversed EIEC infection resulting in a centripetal retraction of the peri-junctional actin filaments with separation of actins from the apical cellular borders and EIEC-induced rearrangements of Claudin-1, Occludin, JAM-1 and ZO-1 proteins in Caco-2 (Qin et al., 2009).

In summary, the protection of mucosal barrier in piglets by probiotics may be achieved mainly through stimulating secretion of mucin and antimicrobial peptides, promoting intestinal fluid absorption and reducing fluid secretion, and upregulating the expression of intestinal TJs protein. From the research status on the regulatory effects of probiotics on the intestinal chemical and mechanical barriers of postweaning piglets, probiotics play an important role in both prevention and treatment of PWD. On the one hand, probiotics promote the secretion of mucin and antimicrobial peptides and up-regulating the expression of TJs protein to prevent PWD. On the other hand, it can alleviate the damage of the intestinal chemical and mechanical barriers caused by PWD. However, the specific regulatory mechanism of probiotics on the intestinal chemical and mechanical barriers in postweaning piglets still needs further research.

## Probiotics Relieve PWD by Regulating the Intestinal Immunological Barrier

Piglets are exposed to complex microbiota after weaning from environment. The intestinal immune system needs to rapidly identify harmful microorganisms and dietary antigens, and trigger the correct mucosal immune response. However, the intestinal mucosal immune system of piglets does not mature until about two weeks after early weaning (Butler and Wertz, 2012). In recent years, many studies have reported the effect of probiotics on the gut immunity of weaned piglets (**Table 2**). Probiotics (such as *Lactobacillus*, *Bacillus*, yeast, etc.) and their metabolites (such as organic acids, mannan oligosaccharide and β-glucan of yeast cell wall, etc.) seem to act as immune activators, which can trigger the proliferation and differentiation of T lymphocytes and B lymphocytes, and promoting the secretion of a series of cytokines and generating a series of immune responses (Sharma et al., 2010).

**Table 2:** Effects of probiotics on immunity of piglets.

Microorganism Category	Microorganism Name	Treatment	Host Health Influence	Reference
Lactic acid bacteria	*Lactobacillus salivarius*	Porcine intestinal epithelial were stimulated with *Lactobacillus salivarius* (5 × 10^7^ cells/mL)	Improving IFN-β, IFN-λ and antiviral factors expression in PIE cells	(Indo et al., 2021)
	*Lactobacillus delbrueckii*	The piglets were orally administrated with *Lactobacillus delbrueckii* (50 × 10^8^ CFU/mL) at amounts of 1, 2, 3, and 4 mL per animal at 1, 3, 7, and 14 d of age	Increasing the concentration of IgG in serum; promoting the production of anti-inflammatory cytokines IL-4 and IL-10, and reducing the content of pro-inflammatory factor IL-1β	(Li et al., 2019a)
	*Lactobacillus frumenti*	Piglets received a PBS suspension (2 mL, 10^8^ CFU/mL) containing the *Lactobacillus frumenti* by oral gavage once a day during the period of 6–20 days of age prior to early weaning	The level of serum IgG, intestinal sIgA, and IFN-γ were significantly increased	(Hu et al., 2018a)
	Lactobacillus reuteri	Intestinal porcine epithelial cells were treated with ETEC and Lactobacillus reuteri	Inhibited ETEC-induced expression of pro-inflammatory transcripts IL-6 and TNF-α and protein IL-6 and increased the level of the anti-inflammatory cytokine IL-10	(Wang et al., 2016b)
	*Lactobacillus plantarum*	Piglets weaned at 25 d of age were fed basal diet supplemented with *Lactobacillus plantarum* (2 × 10^10^ CFU/day) for 7 days, and then orally challenged with ETEC F4 (10^9^ CFU/mL)	The level of serum TNF-α was significantly increased	(Guerra-Ordaz et al., 2014)
Yeast	*Saccharomyces cerevisiae*	Piglets (20 d of age) were fed with *Saccharomyces cerevisiae* (2 × 10^8^ CFU/mL) for 10 days (10 mL/day)	Increasing the numbers of plasmocyte and lymphoid nodule; promoting the development of PPs and germinal center	(Zhaxi et al., 2020)
	Brewery hydrolyzed yeast	Piglets weaned at 25 d of age were fed basal diet supplemented with 2 g/kg brewery hydrolyzed yeast for 28 days	Increased IgG and IgM antibodies in serum-binding KLH, and increased SRBC agglutination titers	(Molist et al., 2014)
	*Saccharomyces cerevisiae *var. *boulardii* and β-galactomannan	Porcine small intestine epithelial cell was challenged *in vitro* with *Escherichia coli* F4 and then treated with *Saccharomyces cerevisiae *var. *boulardii* and β-galactomannan	Decreased the mRNA ETEC-induced gene expression of pro-inflammatory cytokines TNF-α, IL-6, GM-CSF and chemokines CCL2, CCL20 and CXCL8 on intestinal IPI-2I	(Badia et al., 2012b)
	*Saccharomyces cerevisiae*	Porcine small intestine epithelial cell was challenged *in vitro* with *Escherichia coli* F4 and then treated with *Saccharomyces cerevisiaee*	Inhibited the ETEC-induced expression of pro-inflammatory transcripts IL-6, IL-8, CCL20, CXCL2, and CXCL10, as well as proteins IL-6 and IL-8	(Zanello et al., 2011)
*Bacillus*	*Clostridium butyricum*	Piglets (7.09 ± 0.2 kg) were fed basal diet supplemented with *Clostridium butyricum* (5 × 10^5^ CFU/g) for 15 days and then orally administered with ETEC F4 (1 × 10^9^ CFU/g)	Including myeloid differentiation factor, toll-interacting protein, and B cell CLL/lymphoma 3, in the intestines of ETEC F4-challenged piglets	(Li et al., 2021)
	*Bacillus cereus* var. *Toyoi*	Piglets (14 d of age) were fed basal diet supplemented with *Bacillus cereus* var. *Toyo* (6.5 × 10^5^ CFU/g) and then orally administrated with *Salmonella* (3 × 10^9^ CFU per pig) on d 29	Reduced frequencies of CD8^+^ γδ T cells in the peripheral blood and the jejunal epithelium	(Scharek-Tedin et al., 2013)
Three types of mixed bacteria	*Lactobacillus plantarum, Bacillus subtilis and Saccharomyces cerevisiae*	Weaning pigs basal diet supplemented with 15% fermented soybean meal	The level of serum IgG, IgM and IgA were significantly increased, and autophagy factor LC3B in piglets showed a downward trend	(Zhu et al., 2017b)

When weaned piglets are not infected by pathogens such as *E. coli*, the supplement of probiotics actually activates the immune system of piglets to develop towards a more stable and less vulnerable direction. The supplement of probiotics, to a large extent, helps piglets to establish intestinal immunity response against the invasion of pathogens after weaning without causing severe inflammatory responses. Van Baarlen et al., (van Baarlen et al., 2011) reported that lipoteichoic acid, a cell surface molecule of Lactobacillus plantarum, regulated the activation of extracellular signaling kinase through the TLR signaling pathway, in turn activating NF-κB to regulate the release of Th1 cytokines and subsequent T_reg_ and Th1 development. Lactobacillus rhamnosus can regulate the proliferation of T-lymphocytes and increase the number of CD3+ CD4+ T-lymphocytes in the intestine of early weaning piglets (Shonyela et al., 2020). In addition, probiotics also play a role in the stimulation of antibodies in the gut, particularly slgA, which can inhibit pathogen adherence to IECs. Zhu et al. (2017a) suggested that weaning pigs diet supplemented with *Saccharomyces cerevisia*e increase the content of slgA in intestinal mucosa.

On the other hand, when weaned piglets are infected with pathogens, these pathogen-associated molecular patterns are well recognized by the cells of the immune system that reside within the lamina propria, and their activation results in the release of pro-inflammatory mediators and inflammatory responses (Humphrey et al., 2019). Probiotics can enhance the proliferation and differentiation of intestinal immune cells, and inhibit the expression of pro-inflammatory cytokines and promote the expression of anti-inflammatory cytokines, thus protecting the intestinal tract from damage caused by pathogen-related inflammation. Zanello et al. (2011) reported that *Saccharomyces cerevisiae* inhibited the ETEC-induced expression of pro-inflammatory transcripts IL-6, IL-8, CCL20, CXCL2, and CXCL10. Probiotics tend to increase the plasma level of IL-2, and inhibit pro-inflammatory cytokines (*IL-1* and *IL-18*) expression and promote anti-inflammatory cytokines (*IFN-γ*, *IL-4* and *IL-10*) expression (Ngo and Vo, 2019; Xiang et al., 2020). Wang, et al., (Wang et al., 2016b) suggested Lactobacillus reuteri inhibited ETEC-induced the expression of pro-inflammatory transcripts IL-6 and TNF-α and increased the level of the anti-inflammatory cytokines IL-10. Zhu et al. (2014) found that the amelioration of PWD in piglets by Lactobacillus *rhamnosus* is associated with the generation of lamina propria CD3^+^CD4^+^CD8^−^T cells and the expansion of PPs CD3^+^CD4^−^CD8^−^ and CD3^−^CD4^−^CD8^+^ cells. In addition, Virdi et al. (2019) suggested that a single-gene-encoded monomeric immunoglobulin A(IgA)-like antibody, mVHH-IgA, secreted from Pichia pastoris, prevent the colonization of F4 fimbriae-bearing enterotoxigenic *E. coli* in small intestine of piglets. Lactobacillus plantarum was also reported to partially inhibit F4-triggered expression of inflammatory cytokines IL-8 and TNF-α. The induction of negative regulators of TLRs by Lactobacillus plantarum in IPEC-J2 may be important for the inhibition of inflammatory cytokines and may be mediated through the NF-κB and MAPK pathways (Wu et al., 2016).

Overall, in terms of intestinal immunological barrier, probiotics prevent or mitigate PWD by triggering a range of immunological defense mechanisms, including promoting the production of slgA and inflammatory cytokines, and promoting the differentiation of intestinal immune cells. From the research status on the regulatory effects of probiotics on the intestinal immunological barrier of postweaning piglets, probiotics also play an important role in both prevention and treatment of PWD. Probiotics and their metabolites act as immune activators to activate the immune system of postweaning piglets and increase the body’s resistance to pathogenic bacteria, thereby preventing PWD. Additionally, probiotics protect the intestinal tract from damage caused by pathogen-related inflammation. In production, we recommend selecting appropriate probiotics as immune activators to prevent PWD, which may have better economic effects.

## Regulation Mechanism of Probiotics on Intestinal Barriers of Postweaning Piglets

The surface components of probiotics, such as flagella, pili, surface layer proteins (SLPs), capsular polysaccharide (CPS), lipoteichoic acid, and lipopolysaccharide, constitute microbial-associated molecular patterns (MAMPs). They can specifically bind to pattern recognition receptors (PRRs) such as NOD-like receptors (NLRs) and TLRs (Lebeer et al., 2018; Liu et al., 2020). The underlying mechanisms have been proposed for the probiotic (such as *Lactobacillus rhamnosus* and *Lactobacillus plantarum*) effect on epithelial barrier modulation, involving protein kinase C (PKC)- and mitogen-activated protein kinase (MAPK)-dependent pathways, and inhibition of cytokine-induced epithelial cell apoptosis and damage through a phosphoinositide 3-kinase–AKT-dependent pathway. These and similar studies have provided insights into the mechanisms by which probiotics may affect epithelial barrier function at the molecular level (Seth et al., 2008; Anderson et al., 2010; Bron et al., 2012). *Lactobacillus plantarum* also improve epithelial barrier function by inhibiting the reduction of TJs proteins, and reducing the expression of proinflammatory cytokines induced by ETEC F4, possibly through modulation of TLRs, NF-κB and MAPK signaling pathway (Wu et al., 2016). *Lactobacillus acidophilus* was reported to promote Th1 cell development by inducing the production of Th1 cytokines *via* an IFN-STAT3-NF-κB signaling axis (van Baarlen et al., 2011). Fu et al. (2021) reported that *Clostridium butyricum* improved intestinal chemical and mechanical barriers (up-regulating the gene expression of *pBDs*, JTs protein and mucin*)* of weaning piglets by the TLR-2-MyD88-NF-κB signaling. Prebiotics can promote the proliferation of SCFAs-producing microorganisms in the hindgut of weaning piglets, and then reduce the expression of intestinal proinflammatory factors through the MyD88-NF-κB signaling pathway to improve the intestinal immunological barrier (Tian et al., 2022). In addition, metabolites produced by probiotics, such as secreted proteins, indole and SCFAs, also protect the intestinal barriers. Butyrate secreted by *Clostridium butyricum* can promote the expression of hypoxia-inducible factor (HIF-1α) to upregulate the expression of its targeted downstream intestinal JTs, mucin and antimicrobial peptides, and promote intestinal IL-22 secretion, thereby improving the gut barrier and immune function (Pral et al., 2021).The indole-3-lactic acid produced by *Bifidobacterium infantis* and *Lactobacillus reuteri* activates the aryl hydrogen receptors (AhRs) of the gut epithelium by increasing their nuclear localization and up-regulating the protein expression of CYP1A1. The activation of AhRs then leads to lL-22 transcription, which can further increase the expression of antimicrobial peptides (Ehrlich et al., 2018; Hou et al., 2021). The soluble proteins P40 and p75 isolated from *Lactobacillus rhamnosus* can activate EGFR and then up-regulate the expression of an A proliferation-inducing ligand (APRIL) in the epithelium, thus stimulating the secretion of lgA by B cells. Besides, P40 and p75 can activate EGFR–PIK3–Akt signaling pathway to maintain gut homeostasis (Wang et al., 2017).

In summary, the surface molecules and metabolites of probiotics may modulate postweaning piglets’ gut barrier function *via* multiple signaling pathways. Direct use of the surface components and metabolites of probiotics may be considered to prevent or treat PWD instead of probiotics during weaning of piglets.

## Conclusion and Future Perspectives

**Figure 1:**
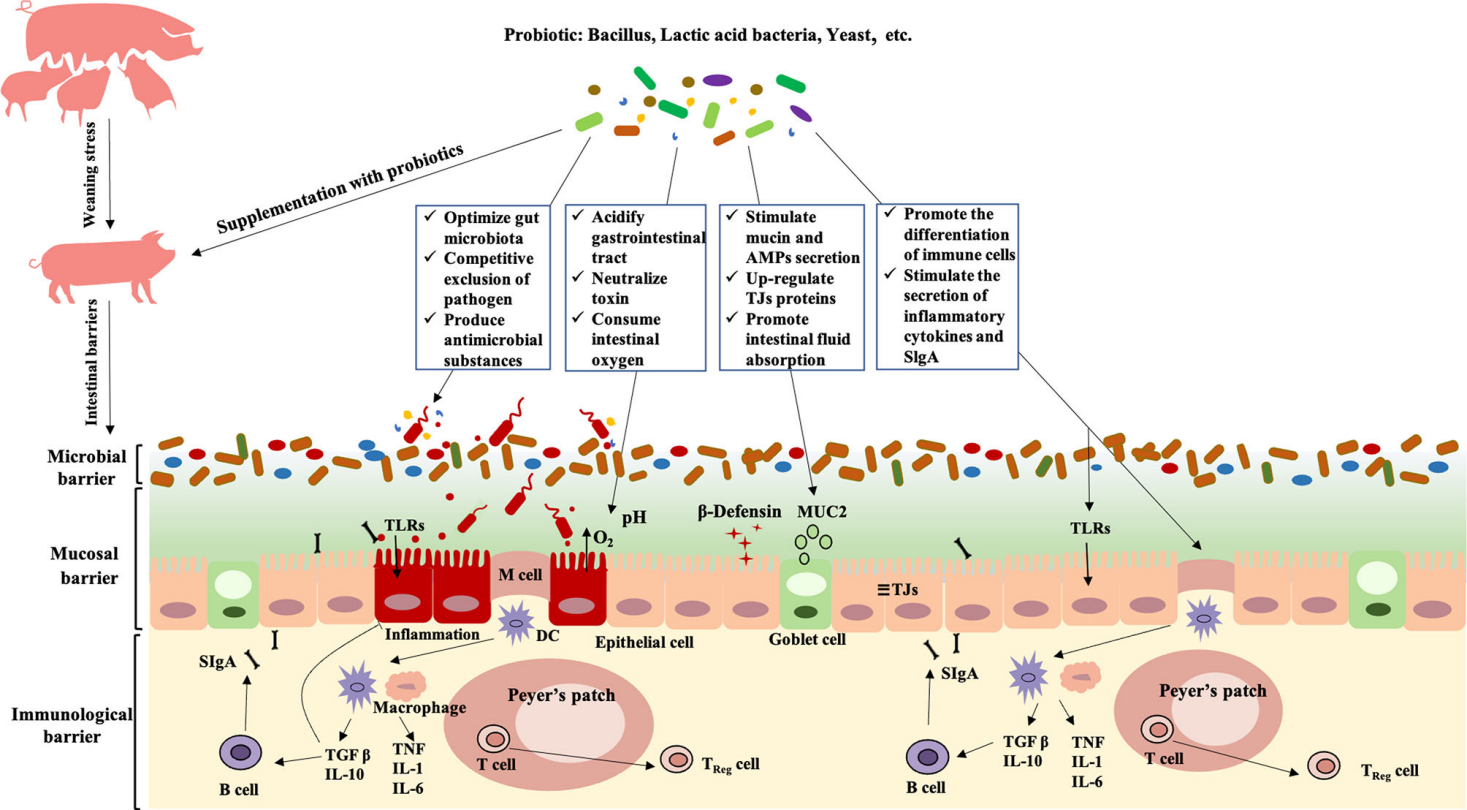
Modulation of probiotics on intestinal barriers in postweaning diarrhea piglets. Probiotics relieve PWD by regulating the intestinal microbial barrier: 1) shaping the gut microbiota oriented by beneficial bacteria; 2) competitive exclusion of pathogen; 3) producing antimicrobial substance and neutralize toxin. Probiotics relieve PWD by regulating the intestinal mucosal barrier: 1) stimulating the secretion of mucin and antimicrobial peptides; 2) upregulation of intestinal tight junction protein expression; 3) maintaining normal intestinal permeability, and promoting intestinal fluid absorption and secretion reduction. Probiotics relieve PWD by regulating the intestinal immunological barrier: 1) promoting the proliferation and differentiation of intestinal immune cell; 2) stimulating the secretion of inflammatory and SlgA. TLRs, Toll-like Receptors; MUC2, Mucin 2; TJs, Tight Junctions; DC, Dendritic Cell; TGF-β, Transforming growth factor-β; TNF, Tumor Necrosis Factor); IL, Interleukin; SIgA, Secretory Immunoglobulin A;AMPs, antimicrobial peptides.

Probiotics is a potential alternative to antibiotics for the prevention and treatment of PWD. We review the research status of PWD-preventing and treating probiotics and discuss its potential regulatory mechanism from the perspective of intestinal barriers. Different from antibiotics, probiotics generally play a role in PWD through restoring the balance of intestinal microecology and regulating intestinal mucosal and immunological barriers. Different probiotic species exert their health-regulatory effects for PWD through diverse ways such as competitive exclusion of pathogen, producing antimicrobial substance and neutralizing toxin, improving intestinal permeability, and promoting the proliferation and differentiation of intestinal immune cell (**Figure 1**). Consequently, probiotics have unique advantages and considerable potential in application to PWD of piglets.

From the research status on the regulation effect of probiotics on the intestinal barriers of postweaning piglets, prevention and treatment combinations may be future directions. More *in vivo* or *in vitro* experiments should be carried out to screen for probiotics (such as *Lactobacillus*) from normal weaned healthy piglets that can stably colonize the piglet’s gut, efficiently produce antibacterial substances, competitively exclude of pathogen, improve intestinal mucosal barrier and activate the immune system to prevent PWD. Although some fecal microbiota transplantation (FMT) experiments have been carried out and achieved good results (Ma et al., 2021), the effect of specific flora or strains on intestinal colonization of early piglets still needs further research. In addition, supplementing probiotics to accelerate the maturation of gut microbiota or to shape a PWD-preventing gut microbiota in weaned piglets are worthy of further study. Furthermore, probiotics may be used as a restorative agent to restore the disorders of gut microbiota and immune system that caused by antibiotics in piglets. Probiotics (prevention) - antibiotics (treatment) - probiotics (repair) may be a new combination strategy for early weaning piglets. Additionally, regulation mechanism of probiotics on intestinal barriers by the surface molecules and metabolites of probiotics suggested that direct use of the surface components and metabolites may be a viable and efficient strategy to prevent or treat PWD instead of probiotics during weaning of piglets.

## Author Contributions

WS: Conceptualization, Writing - original draft. TG and ZJ: Writing - review & editing. ZL and YW: Resources, Writing - review and editing, Supervision. All authors edited, critically revised, and approved the final manuscript. All authors contributed to the article and approved the submitted version.

## Funding

The authors thank the specialized research fund from China Agriculture Research System of MOF and MARA (CARS-35), National Center of Technology Innovation for Pigs, Major Science and Technology Projects of Zhejiang and Shandong (2021C02008, 2019JZZY020602, 2019C02051, CTZB-2020080127).

## Conflict of Interest

The authors declare that the research was conducted in the absence of any commercial or financial relationships that could be construed as a potential conflict of interest.

## Publisher’s Note

All claims expressed in this article are solely those of the authors and do not necessarily represent those of their affiliated organizations, or those of the publisher, the editors and the reviewers. Any product that may be evaluated in this article, or claim that may be made by its manufacturer, is not guaranteed or endorsed by the publisher.
